# The association between insurance coverage for insulin pen needles and healthcare resource utilization among insulin-dependent patients with diabetes in China

**DOI:** 10.1186/s12913-018-3095-9

**Published:** 2018-04-24

**Authors:** Linong Ji, Arthi Chandran, Timothy J. Inocencio, Zilin Sun, Qifu Li, Guijun Qin, Zheng Wei, Stefan DiMario, Richard H. Chapman

**Affiliations:** 1 Endocrinology, Peking University People’s Hospital, Beijing, China; 2 Health Economics and Outcomes Research, BD, 1 Becton Drive #J315b, Franklin Lakes, NJ 07417 USA; 3 Avalere Health, Washington, DC USA; 4 Endocrinology, Southeast University Zhongda Hospital, Nanjing, China; 5 Endocrinology, Chongqing MU Affiliated No. 1 Hospital, Chongqing, China; 6 Endocrinology, The First Affiliated Hospital of Zhengzhou University, Zhengzhou, China; 7 Medical Affairs, BD and Company, Beijing, China; 8 BD, Lakes, Franklin, NJ USA

**Keywords:** Diabetes mellitus, Insulin, Costs, Lipohypertrophy, China

## Abstract

**Background:**

Pen needles are an important component of insulin delivery among patients with diabetes, but are not universally covered in China. We compared clinical and economic characteristics of insulin-dependent patients in China who have some level of pen needle (PN) reimbursement to those with no PN reimbursement.

**Methods:**

A cross-sectional study was conducted among 400 insulin users with Type 1 or Type 2 diabetes treated in outpatient endocrinology units of four large tertiary care hospitals in Nanjing, Chongqing, Beijing and Zhengzhou. Demographics, medical history, healthcare resource utilization (RU), out-of-pocket costs, insurance and PN reimbursement status were surveyed. Unit costs were assigned to healthcare RU and compared using descriptive statistics and multivariate regression models.

**Results:**

A total of 400 patients were analyzed; 142 (35.5%) with some level of PN coverage/reimbursement and 258 (64.5%) without. Patients without PN reimbursement had a higher prevalence of lipohypertrophy (59.3% vs. 40.7%, *p* = 0.0007), greater median PN reuse (12 vs. 7 times per needle, *p* < 0.0001), greater 6-month insulin costs (1591 vs. 1328 Renminbi [RMB], *p* = 0.0025) and total unadjusted 6-month expenditures (6433 vs. 4432 RMB, p < 0.0001), respectively. After controlling for clinical and demographic characteristics, patients without PN reimbursement had 4.6 times greater odds of high costs compared to those with PN reimbursement.

**Conclusions:**

Insulin users without PN reimbursement may pose a greater economic burden to China compared to those with PN reimbursement. Expansion of insurance coverage for insulin PNs can improve the quality of care and potentially help reduce the economic burden in this population.

## Background

Approximately 114 million or 12% adults in China are diagnosed with diabetes and an additional 493 million have pre-diabetes [1]. Many patients with diabetes in China face challenges in accessing adequate care. Available statistics suggest that only 25.8% of patients with diabetes in China have ever received treatment for their diabetes, among whom only 39.7% have achieved adequate glycemic control [Error! Bookmark not defined.]. Diabetes also confers a significant economic burden on China, with direct medical costs for type 2 diabetes mellitus estimated to be US $26.0 billion in 2007 and projected to reach US $47.2 billion in 2030 [2]. Insulin injections are the mainstream treatment for patients with type 2 diabetes in China [3]. Most patients use pens and pen needles (PNs) for injecting insulin, which are intended for single use only. PNs reuse may lead to lipohypertrophy, which could in turn impair absorption of insulin and treatment efficacy [4–6]. Despite the fact that PNs are a central component of diabetes management in China, insurance coverage policies for PNs is suboptimal and varies considerably across the country.

Most individuals with health insurance in China are enrolled in one of the three government-funded programs, namely Urban Employee Medical Insurance, Urban Resident Medical Insurance, and New Rural Cooperation Medical Insurance [7]. As the names suggest, the eligibility for these programs depend on urban versus rural residency and employment history. While the specifics about these three insurance programs are beyond the scope of this paper, in general the Urban Employee Medical Insurance provides the most generous coverage among the three, followed by the Urban Resident Medical Insurance and the New Rural Cooperation Medical Insurance which mainly provides catastrophic coverage for medical expenses. Coverage policies for PNs not only differ by insurance type but may also vary by geographic region. For instance, patients with diabetes enrolled in the Urban Employee Medical Insurance in Nanjing pay between 5%–30% coinsurance (depending on age and setting of care) for PNs while their counterparts in Beijing pay 100% of the costs for PNs out of pocket (OOP).

Little is known about the characteristics of patients with diabetes in China and how insurance coverage policies for PNs may affect their health outcomes and healthcare resource utilization. To address this knowledge gap, we performed a secondary analysis of survey data collected among a sample of patients with diabetes regarding their insurance coverage for PNs and its associations with PNs reuse and healthcare resource utilization and costs.

## Methods

### Study population

This is a secondary analysis of data collected from a previous worldwide survey concerning Injection Technique Practices where 31% of insulin-injecting patients in China indicated they had seen “small bumps or swelling at injection sites.” The study population of interest included adult patients with type 1 or type 2 diabetes who have been self-injecting insulin for at least 1 year. Patients were eligible for the study if they met the following criteria: 1) current insulin users with type 1 or type 2 diabetes with more than one year of continuous use of insulin at the time of study enrollment; 2) age of 18–80 years and body mass index (BMI) of at least 18.5 kg/m^2^; 3) used both an insulin pen and PN; 4) ability to understand the investigator’s questions and complete the study questionnaire; 5) and ability to understand and provide consent to the study and its procedures. Patients with mental disorders, a history of surgical operation or trauma on insulin injection sites, skin disorders or imperfections/anomalies on injection sites, use of insulin pump or syringe, diseases that render the survival of the blood cells or interpretation of HbA1c unreliable, or otherwise not appropriate for observation in the study were excluded. Details about the study population and sample estimation have been previously published [8].

Patient recruitment took place between December 2, 2013 and January 27, 2014 at four endocrinology clinics in Beijing, Nanjing, Chongqing, and Zhengzhou, respectively. Each clinic is affiliated with a large tertiary care hospital (i.e., bed capacity exceeding 500), which in China are the primary providers of care for patients with complex comorbidities. Patients who visited the endocrinology clinics were first invited to participate in the eligibility screening. Eligible patients were subsequently invited to participate in the study. Written consent was obtained from each study participant.

### Data collection

Each patient participated in a face-to-face session with one of the investigators, during which the following clinical information was collected: 1) presence of hyperlipidemia, cardiovascular diseases and other comorbidities; 2) diabetes-related complications; 3) assessment of patient’s injection rotation technique. Patients who rotated between different injection sites and moved the injection point at least 1 cm away from the prior injection point within the same injection site were considered to have proper site rotation; and 4) presence, size, and number of lipohypertrophy nodes. All the investigators were trained to conduct the session in a consistent and unbiased manner. Each participant also underwent a limited physical examination, including measurement of height, weight, waistline, hipline, heart rate, blood pressure, and HbA1c testing.

In addition, each participant completed a self-administered questionnaire to report the following information: 1) demographics (age, gender, education, income, employment); 2) type of insurance coverage and coinsurance amount for PNs; 3) diabetes management (diet, exercise, experience of hypoglycemia in the past 6 months); 4) insulin treatment (brand of insulin, dosing schedule, length of needle used for injections, and injection sites and size of the each site); 5) PNs reuse; 6) receipt of instructions for injections in the past 6 months; 7) diabetes-related healthcare resource utilization in the past 6 months (outpatient or emergency room visits, hospitalization); and 8) estimated out-of-pocket spending on PNs, insulin syringes, insulin syringe needles in the past 4 weeks and indirect costs for time spent by caregivers to support patients with tasks that they cannot complete due to their diabetes, including child care, house work, yard work and other activities of daily living. If missing or abnormal values were observed in the questionnaire, the study staff attempted to contact the patient for clarification. Please reference supplementary material for the case report form.

### Measurement of healthcare resource utilization and costs

Unit costs for healthcare resource utilization were obtained from public and private sources. Unit costs for insulin (2015 RMB) were obtained from IMS MIDAS data and estimated at 0.25 RMB [9]. Unit costs for outpatient and hospital services were obtained from the 4th China National Health Services Survey [10] and were inflated to 2015 RMB using the consumer price index for medical goods in China and were estimated at 374.1 RMB and 6581 RMB, respectively [11]. Because only 6 respondents in the entire sample reported having any emergency department visit during the recall period, those costs were not reported separately. Daily insulin costs and the four-week patient OOP spending were converted to 6-month costs by multiplying the daily insulin costs by a factor of 182 and four-week patient OOP spending by a factor of 6.5. Total 6-month costs were calculated for patients by summing the total costs for outpatient visits, hospitalizations, insulin costs and patient OOP spending for PNs. All costs were inflated to 2015 RMB. Indirect costs were calculated according to average hourly wages, based on urban or rural status, multiplied by self-reported unpaid hours spent on professional services as described in the data collection section.

### Statistical analysis

Demographic and clinical characteristics were compared descriptively between patients with and without insurance coverage for PNs. Pearson’s χ^2^ test or Fisher’s exact test (when cells with *n* < 5 are present) was used to evaluate differences in categorical variables between the two groups, while the Wilcoxon Rank Sum test was performed to assess differences in continuous variables.

Because the same unit costs were applied for each patient, it was likely that evaluation of the total costs in a regression model would result in artificially low variances and increased Type I error. To reduce the potential for this bias, the effect of PN reimbursement on total costs was evaluated using a logistic regression model that dichotomized patients into lower- and higher-cost patients. Patients with total 6-month costs at the 75th percentile or above were categorized as having “high” costs while those with total costs below the 75th percentile were classified as having “lower” costs. This cutoff was determined through an evaluation of the distribution of the data, which suggested that costs for these patients increased substantially upon reaching the 75th percentile. Age, sex, education level, insurance, income, type of diabetes, duration of diabetes, duration of insulin use, frequency of hypoglycemia in the prior 6 months, body mass index, and presence of cardiovascular disease, hyperlipidemia, retinopathy, nephropathy, neuropathy, presence of other complications were included as covariates in the logistic regression. Statistical Analysis Software (SAS) Version 9.3 (SAS Institute, Inc., Cary, NC, USA) was used for all the statistical analyses.

## Results

### Demographic statistics

Of 401 survey completers, one participant was excluded for not providing reimbursement status. A total of 400 eligible patients were included in the study, among whom 142 or 35.5% had medical insurance that covered a portion of PNs costs. Compared to those who paid 100% out of pocket for PNs costs, patients with coverage were slightly older (62.4 vs. 58.0 years, *p* = 0.0002), more likely to be enrolled in Urban Employee Medical Insurance, and had higher income (Table 1).

**Table 1:** Demographic Characteristics, by PN Reimbursement Status

Characteristics	Patients with PN Reimbursement (*N* = 142)	Patients without PN Reimbursement (*N* = 258)	*p*-value*
Patient age (years)			
Mean (SD)	62.4 (9.6)	58.0 (12.2)	0.0002
Median (Q1, Q3)	63 (57, 70)	59.5 (50, 67)	
Sex^†^			
Male (%)	65 (45.8)	135 (52.3)	0.2518
Female (%)	77 (54.2)	123 (47.7)	
Education level^†^			
Primary school level or below (%)	19 (13.4)	30 (11.6)	0.2204
Junior school level (%)	51 (35.9)	75 (29.1)	
High school level (%)	31 (21.8)	68 (26.4)	
Bachelor’s degree (%)	38 (26.8)	80 (31.0)	
Master’s degree or above (%)	1 (0.7)	5 (1.9)	
Other (%)	2 (1.4)	0 (0.0)	
Type of medical insurance^a,b^			
Urban employee medical insurance (%)	123 (87.2)	104 (41.1)	< 0.0001
Urban resident medical insurance (%)	12 (8.5)	66 (26.1)	
New rural cooperation medical insurance (%)	1 (0.7)	48 (19.0)	
Commercial insurance (%)	0 (0.0)	2 (0.8)	
Free medical service (%)	2 (1.4)	24 (9.5)	
Other (%)	0 (0.0)	3 (1.2)	
More than 1 type (%)	3 (2.1)	6 (2.4)	
Income^a^			
No income (%)	3 (2.1)	30 (11.6)	0.0002
Below 1000 RMB (%)	4 (2.8)	16 (6.2)	
1001–3000 RMB (%)	92 (64.8)	109 (42.3)	
3001–5000 RMB (%)	35 (24.7)	71 (27.5)	
5001–10,000 RMB (%)	6 (4.2)	25 (9.7)	
10,001–25,000 RMB (%)	1 (0.7)	4 (1.6)	
Above 25,000 RMB (%)	1 (0.7)	3 (1.2)	

*Q1* Lower 25th percentile, *Q3* upper 25th percentile, *SD* Standard deviation

*P-values were obtained using the χ^2^ test, with the exception of education level and type of medical insurance where the Fisher Exact test was used; *p*-values < 0.05 were considered to be statistically significant

^a^Percentages represent column percentages

^b^6 observations were missing responses for medical insurance

### Clinical characteristics

Compared to those without insurance coverage for PNs, a higher percentage of patients with insurance coverage for PNs had type 2 diabetes versus type 1 diabetes (97.2% vs. 91.4%, *p* = 0.026), cardiovascular disease (61.3% vs. 39.9%, *p* < 0.0001) and hyperlipidemia (54.2% vs. 18.6%, *p* < 0.0001) (Table 2). Lipohypertrophy was more prevalent among those who did not have coverage for PNs (59.3% vs. 41.6%, *p* = 0.0006) than their counterparts who had at least some form of coverage; furthermore, the number of lipohypertrophy nodes was higher in the former group (2 vs. 1 per patient, *p* < 0.0001).

**Table 2:** Clinical Characteristics, by PN Reimbursement Status

Parameter	Patients with PN Reimbursement (*N* = 142)	Patients without PN Reimbursement (*N* = 258)	*p*-value^*^
Type of diabetes^a^			
Type 1 (%)	4 (2.8)	22 (8.6)	0.0260
Type 2 (%)	138 (97.2)	235 (91.4)	
Duration of time with diabetes (years)			
Mean (SD)	11.8 (7.3)	11.8 (7.7)	0.9750
Median (Q1, Q3)	11 (7, 15)	11 (5, 16)	
HbA1c			
Mean (SD)	8.0 (1.5)	8.0 (1.8)	0.7484
Median (Q1, Q3)	7.6 (6.8, 8.9)	7.6 (6.8, 8.8)	
Glucose control^a^			
HbA1c < 7% (%)	43 (30.3)	81 (31.4)	0.8177
HbA1c ≥ 7% (%)	99 (69.7)	177 (68.6)	
BMI			
Mean (SD)	25.1 (3.1)	25.6 (3.2)	0.0951
Median (Q1, Q3)	24.8 (22.7, 27.1)	25.4 (23.5, 27.6)	
Frequency of hypoglycemia in previous six months^a^			
0 (%)	60 (42.3)	104 (40.5)	0.4695
1–2 (%)	42 (29.6)	66 (25.7)	
3+ (%)	40 (28.2)	87 (33.9)	
Duration of insulin therapy (years)			
Mean (SD)	5.9 (5.0)	5.4 (4.3)	0.4444
Median (Q1, Q3)	5 (2, 8)	4 (2, 8)	
Presence of CVD^†^ (% Yes)	87 (61.3)	103 (39.9)	< 0.0001
Presence of hyperlipidemia^a^ (% Yes)	77 (54.2)	48 (18.6))	< 0.0001
Presence of lipohypertrophy^a^ (% Yes)	59 (41.6)	153 (59.3)	0.0007
Number of lipohypertrophy nodes			
Mean (SD)	1.5 (0.7)	2.7 (2.5)	< 0.0001
Median (Q1, Q3)	1 (1, 2)	2 (1, 3)	
Longest diameter of lipohypertrophy nodes			
Mean (SD)	16.8 (18.6)	16.1 (11.9)	0.1224
Median (Q1, Q3)	10 (5, 22)	15 (8, 20)	

*Differences in continuous variables were tested using Wilcoxon Rank Sum tests for non-normally distributed variables and the Student t-test for normally distributed variables; differences in categorical variables were tested using χ^2^ tests; *p*-values < 0.05 were considered significant

^a^Percentages represent column percentages

*CVD* Cardiovascular disease, *HbA1c* Hemoglobin A1c, *Q1* Lower 25th percentile, *Q3* Upper 25th percentile, *SD* Standard deviation

### Pen needle use characteristics

Despite most patients having received prior instructions on injection technique, proper site rotation was suboptimal for both groups. Patients without insurance coverage for PNs reused the needles more frequently than those with pen needle reimbursement (12 vs. 7 uses per needle, *p* < 0.0001) (Table 3).

**Table 3:** PN Re-use and Related Outcomes, by PN Reimbursement Status

Parameter	Patients with PN Reimbursement (*N* = 142)	Patients without PN Reimbursement (*N* = 258)	*p*-value*
Subject rotates inulin injection site^a^ (% Yes)			
Yes (%)	105 (73.9)	212 (82.2)	0.0522
No (%)	37 (26.1)	46 (17.8)	
Subject ever received instruction on insulin injections^a^ (% Yes)			
Yes (%)	131 (92.3)	229 (88.8)	0.2650
No (%)	11 (7.7)	29 (11.2)	
Most recent receipt or review of injection instruction^a^			
Within the past 6 months (%)	14 (10.7)	18 (7.9)	0.9523
Within the past 6–12 months	8 (6.1)	14 (6.1)	
More than 1 year ago	25 (19.1)	47 (20.5)	
More than 2 years ago	35 (26.7)	65 (28.4)	
More than 5 years ago	33 (25.2)	54 (23.6)	
More than 10 years ago	16 (12.2)	31 (13.5)	
Subject re-uses pen needles^a^ (% Yes)	129 (90.9)	251 (97.3)	0.0047
Number of times a single PN is reused by the subject			
Mean (SD)	12.9 (31.1)	19.5 (28.9)	< 0.0001
Median (Q1, Q3)	7 (3, 15)	12 (6, 20)	

*Differences in continuous variables were tested using Wilcoxon Rank Sum tests for non-normally distributed variables and the Student t-test for normally distributed variables; differences in categorical variables were tested using χ^2^ tests; *p*-values < 0.05 were considered significant

^a^Percentages represent column percentages

*Q1* Lower 25th percentile, *Q3* Upper 25th percentile, *SD* Standard deviation

### Healthcare resource utilization and expenditures

Overall, there were minor differences in healthcare resource utilization by PN coverage status (Table 4). Number of diabetes-related outpatient visits were comparable between the two groups (1 vs. 2, *p* = 0.223). A larger percentage of those without PN coverage had at least 1 hospital stay (17.4% vs. 9.1%, *p* = 0.023). Lack of PN reimbursement was associated with greater total daily insulin use (35.0 vs. 29.2 units, *p* = 0.026). After dividing daily insulin doses by the patient’s body weight in kilograms (kg), mean daily insulin dose remained significantly higher for those without PN reimbursement (0.50 vs. 0.45 units/kg body weight, *p* = 0.041).

**Table 4:** Estimated Diabetes and Insulin-related Healthcare Utilization and Expenditures, by PN Reimbursement Status

	Patients with PN Reimbursement (*N* = 142)	Patients without PN Reimbursement (*N* = 258)	*p*-value*
Resource Utilization in Prior Six Months			
Number of Diabetes-related Outpatient Visits During Prior Six Months^a^			
Mean (SD)	2.65 (2.8)	2.50 (2.4)	0.2228
Median (Q1, Q3)	1 (0, 6)	2 (0, 5)	
Any Diabetes-related Hospital Stay During Prior Six Months (% yes)^a^	13 (9.2)	45 (17.4)	0.0234
Daily Insulin Dose (in Units)			
Mean (SD)	29.18 (13.7)	34.97 (20.3)	0.0264
Median (Q1, Q3)	29.5 (18.0, 38.0)	30.0 (20.0, 44.0)	
Daily Insulin Dose per kg of Body Weight			
Mean (SD)	0.45 (0.2)	0.50 (0.3)	0.0414
Median (Q1, Q3)	0.43 (0.28, 0.58)	0.46 (0.28, 0.65)	
Costs in Previous Six Months (reported in 2015 RMB)			
Diabetes-Related Outpatient Costs^b^			
Mean (SD)	1105 (1160.5)	1040 (1006.9)	0.5576
Median (Q1, Q3)	416 (0, 2497)	832 (0, 2081)	
Diabetes-Related Hospital Costs^b^			
Mean (SD)	773 (2717.0)	1589 (4231.8)	0.0388
Median (Q1, Q3)	0 (0, 0)	0 (0, 0)	
Insulin Costs^b^			
Mean (SD)	1328 (624.4)	1591 (922.9)	0.0025
Median (Q1, Q3)	1342 (819, 1729)	1365 (910, 2002)	
Reported OOP Costs			
Mean (SD)	1226 (933.3)	2217 (2079.1)	< 0.0001
Median (Q1, Q3)	995.5 (679, 1580)	1761.9 (891, 2937)	
Total Diabetes-Related Costs			
Mean (SD)	4432 (3376.3)	6433 (5147.2)	< 0.0001
Median (Q1, Q3)	3850 (2135, 5388)	5075 (3441, 7576)	

*Differences in continuous variables were tested using Wilcoxon Rank Sum tests and categorical variables (any diabetes-related hospital stay) using χ^2^ tests; *p*-values < 0.05 were considered significant

^a^Values represent outpatient visits and hospital stays during the past 6 months

^b^Unit costs: 1) insulin costs 0.25 RMB per unit; 2) outpatient/ER visits are 374.1 RMB per visit; 3) and hospital stays are 6581 RMB per stay

*BMI* Body mass index, *DM* Diabetes mellitus, *kg* Kilogram, *OOP* Out-of-pocket, *PN* Pen needle, *Q1* Lower 25th percentile, *Q3* Upper 25th percentile, *SD* Standard deviation

Descriptive comparisons of 6-month healthcare expenditures according to PN reimbursement are provided in Table 4. Patients without PN reimbursement experienced greater hospital expenditures (1589 RMB vs. 773 RMB, *p* = 0.0388), insulin costs (1591 RMB vs. 1328 RMB, *p* = 0.0025), and self-reported OOP costs (2217 RMB vs. 1226 RMB, *p* < 0.0001). The total 6-month standardized expenditures, after excluding patients with missing cost data (1 observation had missing cost data for outpatient costs), were 6433 RMB for patients without PN reimbursement, compared to 4432 RMB (*p* < 0.0001) for those who had PN reimbursement.

After adjusting for demographic and clinical characteristics (Table 5), patients without PN reimbursement had increased odds of having high costs compared to those with PN reimbursement (OR = 4.56, 95% CI = [2.14, 9.75], *p* = < 0.0001). Other factors in the model associated with increased costs included presence of retinopathy (OR = 2.08, 95% CI = [1.13, 3.85], *p* = 0.0195), and presence of neuropathy (OR = 2.92, 95% CI = [1.56, 5.49], *p* = 0.0009).

**Table 5:** Factors Associated with Highest Direct Healthcare Expenditures in Previous Six-Months^a^

Parameter	Odds Ratio	95% CI	*p*-value*
Age (year)	0.95	0.92, 0.98	0.0006
Sex			
Male	0.98	0.55, 1.75	0.9382
Female (ref)	--	--	
Education Level			
High School and Below	0.77	0.37, 1.59	0.4810
Bachelor’s Degree and Above (ref)	--	--	
Type of Insurance			
Urban Employee Medical Insurance*	--	--	--
Urban Resident Medical Insurance	1.72	0.82, 3.63	0.8539
New Rural Cooperation Medical Insurance	1.71	0.65, 4.49	0.8882
Free medical service	1.00	0.32, 3.14	0.3535
Other	2.23	0.31, 16.17	0.6961
> 1 type	2.64	0.50, 14.03	0.4922
Income			
No income (ref)	--	--	--
3000 RMB or below	1.30	0.44, 3.81	0.4037
Above 3000 RMB	0.96	0.28, 3.33	0.6926
Subject has some level of PN Reimbursement			
Yes (ref)	--	--	--
No	4.56	2.14, 9.75	< 0.0001
Type of Diabetes			
Type 1 (%) (ref)	--	--	--
Type 2 (%)	0.64	0.23, 1.85	0.4133
Duration of diabetes	0.96	0.91, 1.01	0.1311
Duration of insulin	1.03	0.95, 1.12	0.4174
Hypoglycemia frequency in previous six months			
None (ref)			
1 to 2	--	--	--
3 or more	1.12	0.56, 2.25	0.5576
	1.84	0.97, 3.48	0.0624
BMI	1.09	1.00, 1.20	0.0558
Presence of CVD	1.84	0.98, 3.48	0.0598
Presence of hyperlipidemia	1.67	0.86, 3.24	0.1300
Retinopathy	2.08	1.13, 3.85	0.0195
Nephropathy	0.90	0.41, 2.00	0.7983
Neuropathy	2.92	1.56, 5.49	0.0009
Other complication	1.03	0.35, 3.05	0.9578

*p-values < 0.05 were considered significant

^a^Total costs under the 75th percentile of costs were considered to be low costs; costs at and above the 75th percentile were considered to be high costs; The cut-off point was based upon the distribution of costs in the data.

*BMI* Body mass index, *CI* Confidence interval, *CVD* Cardiovascular disease, *OR* Odds ratio, *ref.* reference group

## Discussion

Pen needles are an important component of insulin delivery among insulin-requiring patients with diabetes. Despite this, only 35.6% of patients in our sample reportedly had any kind of reimbursement for their PNs (exact coverage could not be verified). This has important implications around patients’ overall care, outcomes and costs. We found that patients who lack PN reimbursement may have significant unmet needs (compared to those who have their PNs reimbursed). These patients had a higher prevalence of lipohypertrophy and increased hospitalizations, insulin use, and overall costs. Although these associations do not indicate causality, they nonetheless indicate that these patients represent a population where improvements in treatment are needed to improve their outcomes and decrease overall costs.

We observed that patients without PN reimbursement had greater costs compared to those with PN reimbursement, even after controlling for various clinical and demographic characteristics. A large portion of these increased costs is likely attributable to hospitalizations, as significant differences in hospitalization rates were observed in bivariate analyses and hospital costs are greater in scale compared to other costs (i.e., insulin costs). Although not directly attributable to PN reimbursement in this study, increased diabetes-related hospitalization and costs nonetheless indicate that these patients experience greater complications that require more intensive medical care.

Patients without PN reimbursement also had increased insulin costs, as a function of greater insulin utilization. The difference in daily average insulin costs (1.45 RMB per day) amounts to approximately 529 RMB per year. Despite their greater utilization of insulin, patients without PN reimbursement had similar average HbA1c levels as those with PN reimbursement, implying that these patients required more insulin to control their blood glucose. The clinical significance of this association is unclear. One reason why this was observed may be due to an increased observed prevalence of lipohypertrophy among these patients.

Despite most patients in the study reporting they had received injection instruction at some point in their lives, only 16.8% received instruction in the year prior. Proper site rotation, as defined by this study to be both site rotation and moving the injection point at least 1 cm away from the prior injection point, was poor overall. However, upon sub-analysis, we found that while patients with PN reimbursement did not move the injection point at least 1 cm away from the prior injection point significantly more often, they did rotate sites significantly more often which was associated with a lower prevalence of lipohypertrophy. The difference in site rotation practices amongst the reimbursed population may allude to a variance in the type of instruction received those reimbursed for PNs. Patients who did rotate generally may have had the intention of proper site rotation, but lack of education on injection technique, or retention, could have undermined their efforts. This finding emphasizes the need for more frequent education on proper injection technique. We also found that more patients without PN reimbursement reused their PNs, and did so more frequently compared to those that had PN reimbursement. PNs are intended for single-use only, yet many patients—especially those without PN reimbursement—reused their PNs. Reuse of PNs has been shown in previous studies to be associated with the development of lipohypertrophy [12, 13]. In particular, one study in Spain among 430 outpatients injecting insulin found that needle reuse greater than 5 times was strongly associated with greater lipohypertrophy [12.]. Another cross-sectional study by Ji et al., conducted in 2010 among 380 diabetes patients across 20 centers in mainland China, also found a significant positive relationship between the frequency of single needle reuse and lipohypertrophy [13]. In the Ji study, the mean number of uses per needle was 9.2, with approximately 26.8% of patients using the PN 10 or more times. Among patients who reused their needles, the most frequent reasons for reusing were for convenience and to save money.

With a growing prevalence of diabetes and use of insulin therapy [1, 14], the lack of reimbursement for PNs may have costly implications. Efforts to improve the quality of care for these patients should be multifaceted, incorporating increased and more frequent patient education, improvements in treatment and monitoring, and implementation of measures to improve patients’ use of prescribed treatment modalities such as PNs.

Evidence suggests that patients’ OOP costs may play a significant role in treatment adherence and clinical outcomes, leading to further potential medical and economic implications.[15] As saving money has been cited as a frequent reason for reusing needles,[13] reimbursement of PNs can help to reduce the overall cost burden on the patient, thereby reducing needle reuse. This may in turn help to reduce lipohypertrophy, which is associated with needle reuse. The healthcare system in China has historically adopted a principle of “broad coverage, with low basic level of benefits”—that is, providing coverage for the greatest number of people with the trade-off of limited levels of benefits. Despite the importance of PNs as a component of diabetes therapy, coverage of PNs has largely been overlooked. Though a larger emphasis is usually placed on drugs rather than medical devices, PNs (or syringes) represent a necessary component for all patients to reliably and safely inject their insulin.

This study has several limitations. First, it is cross-sectional in which we measure both exposures and outcomes at a single point in time. Therefore, although we can observe associations in patient characteristics and outcomes, we cannot evaluate temporal relationships or establish causality on these relationships. Future work should be performed to conduct longitudinal analyses of these outcomes to better understand these relationships over time.

Patients’ healthcare utilization (i.e., outpatient clinic visits and hospitalization) was solicited in the survey via self-report over a recall period of 6 months; this longer period of time may introduce recall bias, in which patients may have difficulty remembering their healthcare utilization during this period, thus resulting in potentially inaccurate estimates of outpatient clinic visits and/or hospitalization. This may be a concern more for minor types of healthcare utilization (e.g., outpatient clinic visits) rather than major events such as hospitalizations. In tradeoff, a shorter time period would increase the risk of not being representative of patients’ healthcare resource utilization.

Total healthcare costs (as opposed to resource utilization) associated with inpatient stays, outpatient visits and insulin use were also not directly solicited from the patient. Therefore, we had to rely on published or private estimates of these costs from the literature or other sources of data. Actual costs may vary widely, especially since different levels of resource intensity may be used depending on the reason for the outpatient visit or hospital stay. Subsequent research should be performed to measure actual healthcare utilization and costs for these patients to shed further insight on the economic burden of these patients.

Many factors can impact patients’ quality of care and outcomes. Disease- and treatment-related factors such as comorbidities, severity of disease, local treatment practices, reimbursement policies for other diabetes-related treatments, and medication adherence can impact patients’ outcomes. We were unable to completely control for these factors, though we were able to control for certain patient comorbidities (such as cardiovascular disease and hyperlipidemia) and complications of diabetes (such as retinopathy, nephropathy, neuropathy, or other complications) as a proxy for severity of disease.

Finally, the studied patient population represents those from endocrinology clinics within four large tertiary hospitals in China, and thus may not be representative of the entire insulin-prescribed diabetes population in China. Larger studies across multiple, geographically-representative centers are needed to better understand the impact of PN reimbursement on health outcomes and costs nationally.

With the limitations being said, this research provides empirical data regarding healthcare costs burden for diabetic patients without PN reimbursement in China. To our best knowledge, this is the first study that explores the differences in economic burden between patients who receive some degree of reimbursement for PNs and those who have to pay 100% out-of-pocket for PNs in China. The work addresses a binary question of whether having some extent of PN reimbursement helps alleviate the economic burden for patients who rely on PN-delivered insulin injections to manage their diabetes. Future research is needed to further evaluate how the degree of reimbursement (i.e., percent of costs reimbursed) may affect the healthcare costs for this patient population, especially those with low income.

## Conclusions

Insulin-dependent diabetes patients without PN reimbursement may confer a larger economic burden on China compared to those with PN reimbursement. To improve outcomes and decrease overall costs, interventions should be considered to improve the quality of care that these patients receive. Further research should focus on illustrating the reasons for hospitalization and increased insulin use among the non-reimbursed population. Providing increased coverage and reimbursement for PNs, along with patient increased awareness of coverage policies, may help to reduce PN reuse and potentially reduce lipohypertrophy and overall healthcare treatment costs for these patients.

## Abbreviations

PN: Pen needle
RU: Resource utilization
RMB: Renminbi
OOP: Out of pocket
BMI: Body mass index
SAS: Statistical Analysis Software
kg: Kilograms
OR: Odds Ratios
CI: Confidence Interval
